# Global Health Security—An Unfinished Journey

**DOI:** 10.3201/eid2313.171528

**Published:** 2017-12

**Authors:** Michael T. Osterholm

**Affiliations:** University of Minnesota, Minneapolis, Minnesota, USA

**Keywords:** infectious diseases, Global Health Security Agenda, global health security

## Abstract

This supplement is a timely, comprehensive compendium of the critical work being done by the Centers for Disease Control and Prevention and various partners to enhance and expand the Global Health Security Agenda. This perspective provides a review of, and comments regarding, our past, current, and future challenges in supporting the Global Health Security Agenda.

*“It’s no use saying, ‘We’re doing our best.’ You have got to succeed in doing what is necessary.” *—Sir Winston Churchill (*1*)

We have witnessed numerous global public health achievements over the past century, resulting in major gains in life expectancy. These achievements resulted primarily from our unprecedented ability to prevent and control infectious diseases. Because of technological advances, such as electricity, we were able to provide safe water and sewage systems (*2*). We manufactured vaccines and antimicrobial drugs and, in some situations, stored and distributed them via reliable cold chains around the world. We began to refrigerate our pathogen-vulnerable food. Pasteurization of milk supplies became commonplace. Smallpox eradication, the near elimination of *Aedes aegypti* mosquitoes from the Americas, and major gains against killer childhood vaccine-preventable diseases led some to proclaim in the 1970s that we had beaten infectious diseases.

However, as we entered the 1980s, any sense of celebration ended as the HIV/AIDS pandemic took hold and outbreaks of emerging pathogens were increasingly recognized. Key victories began to fade as the growing number of failed states around the world made basic public health activities like vaccination extremely difficult and sometimes dangerous. Furthermore, the more than quadrupling of the human population since 1900, especially skyrocketing growth in megacities of the developing world, and the unprecedented level of global trade and travel (3.6 billion international air passengers in 2016) have ensured that emerging microbial pathogens could navigate the globe quickly. Finally, growing awareness of the looming threat of antimicrobial drug resistance has changed our view about being able to successfully manage and treat many life-threatening infections.

The outbreak of severe acute respiratory syndrome in 2003 was a wake-up call to the global public health community that it lacked an international vehicle for rapidly detecting and responding to a multicountry outbreak, particularly one caused by a respiratory-transmitted agent. Despite the World Health Organization’s (WHO’s) adoption of the International Health Regulations 2005 to address this concern, the 2009 pandemic of influenza A(H1N1) was a “live fire demonstration” that the world was still ill-prepared for global public health emergencies. Subsequent emerging microbial threats, including cholera in Haiti (2010), Middle East respiratory syndrome coronavirus (MERS-CoV) in the Middle East and Korea (2012), chikungunya in 2013 and Zika in 2015 in the Americas, yellow fever in Africa in 2015–2016 and in South America in 2016–2017, and cholera in Yemen (2017), highlight the challenges in accomplishing effective global public health preparedness. Most notably, the Ebola epidemic in West Africa in 2014–2016 provided a case study of our numerous global response deficiencies (*3**–**5*).

What has changed to make the world a safer place against infectious diseases, given the cumulative lessons learned from severe acute respiratory syndrome, influenza A(H1N1), Ebola, and other emerging threats? The Global Health Security Agenda (GHSA) was launched by 29 countries, WHO, the Food and Agriculture Organization of the United Nations, and the World Organisation for Animal Health in February 2014, just as the Ebola outbreak was unfolding (*6*). GHSA is now a growing partnership of more than 60 nations and organizations designed to help build countries’ capacity to elevate global health security. GHSA pursues a multisectoral approach to strengthen global and national capacity to prevent, detect, and respond to human and animal infectious disease threats, whether occurring naturally or accidentally or deliberately spread.

The Centers for Disease Control and Prevention (CDC) supports staff in 35 countries. In 2017, CDC supported work in 49 countries conducting broad-based capacity-building efforts to help ensure global health security. It is critical to consider that although CDC’s mission is to protect Americans, we cannot ensure domestic preparedness without ensuring that global infectious disease threats are contained at the source before they reach the United States. The number of countries that are currently strengthened through these CDC health security programs is, however, dependent on intermittent US government funding. Moreover, the 1-time, 5-year emergency congressional funding in 2014 to end the West Africa Ebola epidemic and implement GHSA in US-supported countries ends in 2019.

This supplement of *Emerging Infectious Diseases* is a timely, comprehensive compendium of the critical work being done by CDC and various partners to enhance and expand global health security. The article by Tappero and colleagues (*7*) presents an overview drawing from several articles in this issue and also provides an excellent historical summary of CDC’s invaluable contributions to global health security. This supplement contains articles on GHSA progress, the Joint External Evaluation process, the recent West Africa Ebola outbreak, and building capabilities in disease surveillance, workforce, emergency response and preparedness, laboratory partnerships, and national public health institutes.

One of CDC’s finest hours in its entire 71-year history was its response to the West Africa Ebola outbreak. Many international organizations responded to the outbreak, including WHO and key nongovernmental organizations, but CDC’s effort, with >3,500 staff deployments, was consequential to bringing the epidemic under control and preventing the emergence of a major outbreak in Nigeria. WHO is the international lead agency for global outbreak response, but CDC’s technical expertise, epidemiologic and laboratory workforce development training, and disease detection programs are cornerstones for ministry of health and WHO health security activities globally.

Will GHSA and WHO’s and CDC’s efforts help create a world safer from infectious disease threats and elevate global health security as a priority? Can the international public health community effectively prevent, detect, and respond to human and animal infectious disease threats? These programs help advance the global agenda for infectious disease prevention and control, but we still need to garner greater political will for additional progress. Recently, in our book *Deadliest Enemy: Our War Against Killer Germs* (*2*), Mark Olshaker and I detailed a 9-point crisis agenda if the world is to minimize, if not eliminate, the risk of catastrophic pandemics, outbreaks of critical regional importance, and intentional use of biologic weapons, including genetically altered pathogens.

At the top of our crisis agenda are 2 frightening scenarios: the rapidly emerging consequences of a 1918-like influenza pandemic and the slow-moving tsunami of antimicrobial drug resistance. Outbreaks of critical regional importance include diseases such as Ebola, Lassa fever, Nipah, MERS, and mosquito-borne diseases like Zika. Finally, the prospect for the intentional use of biologic agents cannot be understated. This scenario is often seen through the lens of the 22 cases of anthrax, including 5 deaths, that occurred on the heels of the September 11, 2001, attacks in the United States. This limited number of cases does not portend the public health crisis this attack triggered and the extensive public health resources required to respond to it. A future, much larger bioterrorism attack with a highly lethal agent, such as drug-resistant *Bacillus anthracis*, variola virus, or some other genetically altered pathogen, is not only possible but also highly likely. For true global health security, governments and philanthropic organizations must support Manhattan Project–like initiatives in research, development, manufacturing, and distribution of game-changing vaccines for high-priority pathogens. The new Coalition for Epidemic Preparedness Innovations is a good start, but we need to greatly expand these and related efforts to quickly address the types of objectives outlined in our crisis agenda. For example, we need a similar initiative for developing new antimicrobial drugs and alternative therapies, like phage treatment, for antimicrobial drug–resistant infections. Point-of-care diagnostics to enhance early appropriate antimicrobial therapy are also urgently needed. 

All countries need to have the laboratory, trained workforce, surveillance, and emergency operations capabilities to prevent, detect, and respond to disease threats. Only when these accomplishments are realized can we truly be on the road to global health security for infectious diseases. Until then, the goal of global health security remains an unfinished journey.
